# Supplementary material for: Changes in precipitation and atmospheric N deposition affect the correlation between N, P and K but not the coupling of water-element in Haloxylon ammodendron
Source: PLoS One. 2021 Oct 22;16(10):e0258927. doi: 10.1371/journal.pone.0258927 (PMC8535465; doi:10.1371/journal.pone.0258927)
Supplement: S1 File — (DOCX) [file pone.0258927.s001.docx]

Supplementary information

**Table S1 Pearson correlation coefficients of the correlations between N, P, K contents and ins-WUE, int-WUE under W0 treatments, W1 treatments, N0 treatments, N1 treatments, N2 treatments N+W0 treatments and N+W1 treatments.**

| Elemental contents | ins-WUE | int-WUE | Elemental contents | ins-WUE | int-WUE |
| --- | --- | --- | --- | --- | --- |
| N (W0) | -0.130^ns^ | -0.154^ns^ | N (N0) | 0.113^ns^ | 0.038^ns^ |
| P (W0) | 0.149^ns^ | 0.136^ns^ | P (N0) | 0.308^ns^ | 0.331^ns^ |
| K (W0) | 0.054^ns^ | 0.091^ns^ | K (N0) | 0.591^ns^ | 0.664^ns^ |
| N (W1) | -0.229^ns^ | -0.206^ns^ | N (N1) | -0.388^ns^ | -0.397^ns^ |
| P (W1) | 0.168^ns^ | 0.207^ns^ | P (N1) | 0.188^ns^ | 0.174^ns^ |
| K (W1) | 0.046^ns^ | 0.130^ns^ | K (N1) | -0.149^ns^ | -0.023^ns^ |
| N (N+W0) | 0.198^ns^ | 0.146^ns^ | N (N2) | -0.268^ns^ | -0.250^ns^ |
| P (N+W0) | 0.298^ns^ | 0.212^ns^ | P (N2) | 0.104^ns^ | 0.155^ns^ |
| K (N+W0) | -0.093^ns^ | -0.028^ns^ | K (N2) | -0.028^ns^ | 0.019^ns^ |
| N (N+W1) | -0.678^ns^ | -0.664^ns^ | - | - |  |
| P (N+W1) | 0.019^ns^ | 0.139^ns^ | - | - |  |
| K (N+W1) | -0.011^ns^ | 0.096^ns^ | - | - |  |

Note: ^ns^ represents no significant correlation between two variables. W0 treatments include W0N0, W0N1 and W0N2; W1 treatments include W1N0, W1N1 and W1N2; N0 treatments include W0N0 and W1N0; N1 treatments include W0N1, and W1N1; N2 treatments include W0N2 and W1N2 treatment; N+W0 include W0N1, and W0N2; N+W1 include W1N1, and W1N2.

**Table S2 Pearson correlation coefficients of the correlations between gas exchange and elemental contents in *H. ammodendron***

| Elemental contents | Photosynthetic rate (A) | Stomatal conductance (g_s_) | Transpiration rate (E) |
| --- | --- | --- | --- |
| N | 0.062^ns^ | 0.102 ^ns^ | 0.128 ^ns^ |
| P | 0.223 ^ns^ | 0.069 ^ns^ | 0.074 ^ns^ |
| K | 0.175 ^ns^ | 0.006 ^ns^ | 0.043 ^ns^ |

Note: ^ns^ represents no significant correlation between two variables.

**Table S3 Variations in N/P and N/K across treatments.**

| Treatment | N/P | N/K |
| --- | --- | --- |
| W0N0 | 19.45 ± 0.94^a^ | 0.84 ± 0.12^b^ |
| W0N1 | 21.64 ± 1.36^a^ | 0.99 ± 0.12^b^ |
| W0N2 | 19.99 ± 0.32^a^ | 1.07 ± 0.06^ab^ |
| W1N0 | 19.95 ± 2.81^a^ | 0.94 ± 0.13^b^ |
| W1N1 | 23.60 ± 1.97^a^ | 1.38 ± 0.11^a^ |
| W1N2 | 19.89 ± 1.17^a^ | 1.05 ± 0.12^ab^ |

Note. Data are the mean value ± standard error (SE). Different lowercase letters indicate significant differences across treatments by LSD (least significant difference) test (p < 0.05).
