# Supplementary material for: Changes in precipitation and atmospheric N deposition affect the correlation between N, P and K but not the coupling of water-element in Haloxylon ammodendron
Source: PLoS One. 2021 Oct 22;16(10):e0258927. doi: 10.1371/journal.pone.0258927 (PMC8535465; doi:10.1371/journal.pone.0258927)
Supplement: Attachment — Submitted filename: Comments to the Author.docx [file pone.0258927.s005.docx]

Comments to the Author

Review of the manuscript entitled “Precipitation change and atmospheric N deposition affect the correlation between N, P and K but not the coupling of water-element in H. ammodondron” by Chen et al.

The manuscript titled: “Precipitation change and atmospheric N deposition affect the correlation between N, P and K but not the coupling of water-element in H. ammodondron” by Chen et al. presents interesting results on the Gurbantunggut Desert. But there are a few significant limitations that need to be addressed in a revised version of this manuscript.

1. The whole paper needs a thorough edit (and spelling check) by an English first-language speaker before resubmission.
2. Results and Discussion: Your paper has two sections such as “Results and discussion”, so you should just represent the results with concise and precise sentences and then take further discussion in the discussion section. But in your paper, lots of discussion sentences appear your results section. Please cancel all information related to discussion in your results section
3. **Specific comments：**

Line 49 water is the Plant water economy and nutrition economy? please define it clearly, and early, in the introduction.

Line 55-56 I think references are needed here.

Line 77-81 is there a direct logical relationship between the two ecosysytems?

Line 106 suggest to revises “woods” to “trees”

Line 136 suggest to delete “Due to no leaves,” I think this is an inaccurate statement.

Line 156 suggest to delete “which was its prime assimilating organ”

Line 156 what is “slight positive”? It is unclear

Line 156 How to understand “no relationships between…….” I think this is an inaccurate statement

Line 264-266 Please, check the language in this statement. It is unclear
