# Supplementary material for: Changes in precipitation and atmospheric N deposition affect the correlation between N, P and K but not the coupling of water-element in Haloxylon ammodendron
Source: PLoS One. 2021 Oct 22;16(10):e0258927. doi: 10.1371/journal.pone.0258927 (PMC8535465; doi:10.1371/journal.pone.0258927)
Supplement: Attachment — Submitted filename: responses to the review-3.docx [file pone.0258927.s006.docx]

**Reviewer 1**

**Title: I think the name of the studied plant should be in full spell.**

**Response:**

Thank you for your suggestion! We have revised the title. Please see line 3 at the Revised Manuscript with Track Changes.

**Introduction:**

**L55 “…, which are affected by plant N…” I don’t quite understand what did this “which” refer to, WUE or gas exchanges? Also for this sentence L55-57 I think the authors should explain further why the WUE (or water use) is expected to be related to plant nutrient contents. This is the basis and significance of understanding the results of this study. I see that the authors tried to bring this issue in L60-62, but the explanation was not clear.**

**Response:**

Thank you for your comment! We have revised our writing and added the reason why WUE is expected to be related to plant N, P and K. Please see line 56-68 at the Revised Manuscript with Track Changes.

**L66-68 Here the authors mentioned that “little attention has been paid to the influence of N deposition and precipitation change on these relationships”. However in the next paragraph, “the effects of rainfall change and N deposition on these couplings have been observed in grassland and forest ecosystems [22,23]” (L77-79). I think the current progresses on this topic should be properly introduced in the section.**

**Response:**

Sorry! Our writing may mislead you. From line 74 to line 108 at the Revised Manuscript with Track Changes, we first put forward the main concern in our article, which is also the problem that this article will solve. Then around this concern, we made 3 hypotheses. **(1)** The main concern is that little attention has been paid to the influence of N deposition and precipitation change on these relationships between WUE and plant N, P, K, thus, there is great uncertainty about this issue; although previous studies have reported that N deposition and precipitation change have resulted in the variation of plant N/P ratio and N/K ratio, which indirectly confirmed that plant element coupling was affected by the changes in rainfall and N deposition (see lines 74-89). **(2)** The coupling between WUE and plant N, P, K and the coupling between plant elements have been found in grassland and forest ecosystems, we made the first hypothesis that these couplings should also occur in H. ammodendron (see lines 96-100). **(3)** The effects of rainfall change and N deposition on these element couplings have been observed in two meta-analyses, thus, we made the second hypothesis that the correlation between plant nutrients should also vary with changes in rainfall and atmospheric N deposition for *H. ammodendron* (see lines 100-104). (4) The respond of elements to N deposition and precipitation may vary across elements, and WUE is influenced by N deposition and precipitation; therefore we made the third hypothesis that changes in rainfall and atmospheric N deposition also have an effect on these correlations between WUE and N, P, K (see lines 104-108).

**Overall this section lacks a clear review on the current knowledge of the coupling between plant WUE and nutrient status (related to absorption and utilization) and its response to global change factors.**

**Response:**

Thank you for your comment! We have added the review on the current knowledge of the coupling between plant WUE and nutrient status (related to absorption and utilization) and its response to global change factors, especially precipitation and atmospheric N deposition. Please see the Introduction section (Lines 56-68, 80-89 at the Revised Manuscript with Track Changes).

**Results:**

**In the experiment there were two factors, that is, water addition and N addition. However, the authors only used ANOVA to analyze the effects of different treatments on plant WUE, nutrients and soil variables. As such, the effects of watering, N addition and their interactions on these variables were not statistically assessed. In addition, the authors used a lot of “slightly” or “slight” in describing the changes in certain variables by treatments of watering or N addition. This is not appropriate because it cannot reflect the significance of the statistical tests. The description of results should be precise about whether the change is significant or not. Words like “slightly higher” should be revised unless the authors feel a strong need to describe the result that way.**

**Response:**

Thank you for your suggestions. Based on these suggestions, we have made a big change. We have conducted a two-way ANOVA analysis to assess the effects of watering, N addition and their interactions on plant N, P, K and soil N, P. Please see lines 216-233 and Table 1 at the Revised Manuscript with Track Changes. In addition, we have simplified the expression of the results, and deleted many verbose and meaningless expressions.

**L223-225 It is my feeling that this subtitle, as well as the next ones in L252-253 and L273-275 are too long.**

**Response:**

Thank you for your comment. We have revised these subtitles. Please see lines 244-246, 273-275 and 297-299 at the Revised Manuscript with Track Changes.

**L273-275 This subtitle is confusing. Does it means that here the authors want to present the correlations of data from all treatments?**

**Response:**

Sorry for our poor writing. In this sub-section, we presented the correlation between WUE and N, P, K and the correlation between plant N, P, K under two conditions: adding N and no adding water (W0 + N treatments, includes W0N1 and W0N2) and adding both N and water (W1 + N treatments, includes W1N1 and W1N2). The aim is to assess the effect of the interaction of water and N addition on the correlation between WUE and N, P, K and the correlation between plant N, P, K. We have revised this subtitle. Please see lines 297-299 at the Revised Manuscript with Track Changes.

**Also the figures and their captions can be improved. I understand that the authors are trying to present the different correlations of N-P (and N-K and P-K) as affected by watering or N addition by comparing the slopes (or significance) of the linear regressions. It would be better to present the linear regression lines in a same plot, which may provide a more direct view on the effect of watering or N addition level. For example in Figure 2, for the N-P relationship, the authors may put data from W0 and W1 treatments all in one plot but with different colors, which may help to show the different N-P relationships as affected by watering level.**

**Response:**

Thank you for your suggestion. We have revised the figure, please see Fig. 2-4.

**Discussions:**

**L308-321 I feel that it is better to put these sentences in the Introduction section. Some other sentences in this paragraph are also useful in the Introduction as information of the relationship between plant WUE and nutrient status.**

**Response:**

Thank you for your comment. We have moved these sentences into the Introduction. Please see lines 56-68 at the Revised Manuscript with Track Changes.

**L337-345 Here the authors proposed that the lack of significant coupling between WUE and nutrient contents is related to the slow-growing of H. ammodendron plant. I wonder if there is any special characteristic in leaf or root morphology of this species that helps to survive the desert. These characteristics may also be relevant for the uncoupled plant WUE and nutrient status.**

**Response:**

Your idea is correct. It may be associated with the roots of *H. ammodendron*. They are inserted into the soil layer deeper than 3 m to uptake groundwater (Sheng et al., 2004), so groundwater is an important or most important source of water for the plant species. The N, P, K contents in groundwater may be relatively low. Therefore, even if gas exchanges change, the absorption of these elements by *H. ammodendron* does not necessarily change accordingly, resulting in gas exchanges independent of N, P, K and thus no correlation between WUE and N, P, K. We added the possible mechanism. please see lines 353-360 at the Revised Manuscript with Track Changes.

**L362-366 I would like to see a more precise explanation on the mechanisms of the trade-off strategies that result in the “proportional” and “disproportional” absorption of nutrients by plant.**

**Response:**

Thank you for your comment. The proportional absorption is caused by the proportional requirement of N, P, K by plants. The disproportional absorption may be associated with disproportional availabilities of soil N, P, K induced by adding water and adding N. However, the trade-off strategies of disproportional absorption of N, P and K is temporary. If precipitation and N input increase for a long time, in order to survive, plants will eventually return to a state of absorbing nutrients in proportion. We have added the detailed explanation, please see lines 396-407 at the Revised Manuscript with Track Changes.

**L394-398 This sentence is too long and confusing.**

**Response:**

Thank you for your comment. Since this sentence is verbose and meaningless, we have deleted it. Furthermore, we have deleted S1 Table and S3 Table because these two tables are also meaningless.

**Overall I feel the authors discussed too many on the changes in plant NPK contents with watering and N addition and used it as the explanation of the varied NPK coupling as affected by watering and N addition. There are many literatures on the ecosystem stoichiometry including plant nutrient ratios. I suggest that the authors should consider the underlying mechanisms of theses plant stoichiometric ratios, and make efforts to explain their results based on both plant physiology and ecology aspects.**

**Response:**

Thank you for your suggestion. Based on the suggestion, we made a great revision on the discussion (see lines 374-395 at the Revised Manuscript with Track Changes). We used the knowledges of the physiology of K (lines 376-380) and the ecology theory of P limitation (lines 380-387) to explain our results. In addition, in order to prove that *H. ammodendron* suffered from P limitation, we have added a new S3 Table showing the N/P ratio in *H. ammodendron*.

**Conclusion:**

**L414-415 “… all exerted significant effects …” From what I understand, this statement is based on comparing the slope of linear regressions (or correlation coefficients) under different watering or N addition levels. I don’t see a statistical analysis on the effect of watering, N addition and their interactions on the correlations. So the word “significant” can be misleading.**

**Response:**

Thank you for your suggestion. We have deleted this word. Please see line 428 at the Revised Manuscript with Track Changes.

**Reviewer 2**

1. **The whole paper needs a thorough edit (and spelling check) by an English first-language speaker before resubmission.**

**Response:**

Thank you for your suggestion. We have asked Dr. Eric Posmentier in the department of Earth Sciences, Dartmouth College to edit English.

2. **Results and Discussion: Your paper has two sections such as “Results and discussion”, so you should just represent the results with concise and precise sentences and then take further discussion in the discussion section. But in your paper, lots of discussion sentences appear your results section. Please cancel all information related to discussion in your results section**

**Response:**

Thank you for your suggestion. we have simplified the expression of the results, and deleted many verbose and meaningless expressions and the discussion in the Results Section. Please see lines 216-233, 255-257, 286-289 and 310-312 at the Revised Manuscript with Track Changes.

3. **Specific comments：**

**Line 49 water is the Plant water economy and nutrition economy? please define it clearly, and early, in the introduction.**

**Response:**

Thank you for your suggestion. We have revised our writing. Please see lines 50-51 at the Revised Manuscript with Track Changes.

**Line 55-56 I think references are needed here.**

**Response:**

Thank you for your suggestion. We have added a description of the mechanism behind the correlation between WUE and gas exchange and N, P, K; and references have been cited in this part. Please see lines 56-68 at the Revised Manuscript with Track Changes.

**Line 77-81 is there a direct logical relationship between the two ecosysytems?**

**Response:**

Thank you for your comment. We made a mistake in the previous version. These two previous studies [32,33] (the original version is [22, 23]) are meta-analysis on global scale, not research done in a single ecosystem. We have revised the expression, please see lines 100-102 at the Revised Manuscript with Track Changes. Since the effects of changes in rainfall change and N deposition on the N, P, K couplings have been observed on global scale, we assumed that the correlation between plant nutrients should also vary with changes in rainfall and atmospheric N deposition for *H. ammodendron*. This is the logic of our writing.

**Line 106 suggest to revises “woods” to “trees”**

**Response:**

Thank you for your suggestion. We have revised this word. Please see line 133 at the Revised Manuscript with Track Changes.

**Line 136 suggest to delete “Due to no leaves,” I think this is an inaccurate statement.**

**Response:**

Thank you for your suggestion. We have revised our writing. Please see line 163 at the Revised Manuscript with Track Changes.

**Line 156 suggest to delete “which was its prime assimilating organ”**

**Response:**

Thank you for your suggestion. We have deleted this sentence. Please see line 184 at the Revised Manuscript with Track Changes.

**Line 156 what is “slight positive”? It is unclear**

**Response:**

Sorry. I don’t see “slight positive” in line 156. However, due to this expression is unclear and useless, we have deleted this expression in the whole manuscripts.

**Line 156 How to understand “no relationships between…….” I think this is an inaccurate statement**

**Response:**

Thank you for your suggestion. We have revised our writing. Please see line 251 at the Revised Manuscript with Track Changes.

**Line 264-266 Please, check the language in this statement. It is unclear**

**Response:**

Thank you for your suggestion. Since this sentence may be inapposite. We have deleted it. Please see lines 286-289 at the Revised Manuscript with Track Changes.
