# Supplementary material for: Changes in precipitation and atmospheric N deposition affect the correlation between N, P and K but not the coupling of water-element in Haloxylon ammodendron
Source: PLoS One. 2021 Oct 22;16(10):e0258927. doi: 10.1371/journal.pone.0258927 (PMC8535465; doi:10.1371/journal.pone.0258927)
Supplement: Attachment — Submitted filename: Comments to the Author2.docx [file pone.0258927.s007.docx]

Comments to the Author

This is the second time I have reviewed the manuscript entitled “Precipitation change and atmospheric N deposition affect the correlation between N, P and K but not the coupling of water-element in H. ammodondron” by Chen et al. and I feel the authors have really improved the manuscript and limited the scope of their results to accurately reflect what the data are indicating.but,the standard of English is poor - even in this revision. Providing this is done, the manuscript is in my opinion acceptable for publication.
