# Supplementary material for: Changes in precipitation and atmospheric N deposition affect the correlation between N, P and K but not the coupling of water-element in Haloxylon ammodendron
Source: PLoS One. 2021 Oct 22;16(10):e0258927. doi: 10.1371/journal.pone.0258927 (PMC8535465; doi:10.1371/journal.pone.0258927)
Supplement: Attachment — Submitted filename: Responses to the Reviewers.docx [file pone.0258927.s008.docx]

**Reviewer #1: I see that the manuscript was greatly improved from the old version. There are still some problems, but most of them are minor and easy to correct. In particular I suggest the authors to take care of the use of semicolon. There were quite a lot in the manuscript, which made some sentences too long to read.**

**Response:**

Thank you for your suggestion! We have reduced the use of semicolon.

**Here are my specific comments on the ms:**

**L52: The first appearance of N, P, and K should be in their full spell. In addition, it is better to use their full spell if they are the first words in a sentence, e.g. L61 and L64.**

**Response:**

Thank you for your suggestion! We have revised the spell. Please see lines 54, 55, 57, 65 and 68 at the Revised Manuscript with Track Changes.

**L65: change to “osmotic pressure and enzyme activity [19-21], and thus strongly controls …”**

**Response:**

Thank you for your suggestion! We have revised the sentence. Please see lines 69-70 at the Revised Manuscript with Track Changes.

**L74-77: The first sentence is the same with L48-49, so it is not necessary to state again. Please delete these texts before the semicolon. In addition, I noticed that the authors used quite a lot of semicolons in the text, which has caused the manuscript to be less readable. Please revise.**

**Response:**

Thank you for your suggestion! We have revised the sentence. Please see lines 79-81 at the Revised Manuscript with Track Changes. And we have reduced the use of semicolon as far as possible.

**L82-84: Here is an example of the bad use of semicolon. The sentence should be separated into two sentences for better reading.**

**Response:**

Thank you for your suggestion! We have revised the sentence. Please see lines 88-90 at the Revised Manuscript with Track Changes.

**L88-89: Another semicolon again. In addition, I feel that the two parts have stated the similar meaning. The influence of precipitation and N deposition change on WUE-nutrient relationship is less known, so of course there is uncertainty. But what does this uncertainty mean or imply? I suggest the authors should expand further to explain this uncertainty, otherwise the latter sentence after the semicolon is useless and should be deleted.**

**Response:**

Thank you for your suggestion! We have revised the sentence. Please see line 96 at the Revised Manuscript with Track Changes.

**L96-108: I see that the authors are trying to explain how did these hypotheses come out, but the texts are in fact a repeated of those in the above paragraphs. I suggest to revise these sentences into “****Given the universal plant WUE-nutrient coupling and plant NPK coupling, and the possible impact of changes in precipitation and N deposition on these couplings, we hypothesized that: (1)…; (2)…; (3)….”**

**Response:**

Thank you for your suggestion! We have revised the sentence. Please see lines 104-111 at the Revised Manuscript with Track Changes.

**L108: change to “we** **conduced a field experiment …”**

**Response:**

Thank you for your suggestion! We have revised the sentence. Please see line 122 at the Revised Manuscript with Track Changes.

**L112: “grown” should be “growing”**

**Response:**

Thank you for your suggestion! We have revised the word. Please see line 127 at the Revised Manuscript with Track Changes.

**L113: move “in desert ecosystem” to the end of this sentence.**

**Response:**

Thank you for your suggestion! We have revised the sentence. Please see lines 128-129 at the Revised Manuscript with Track Changes.

**L217: Please check if this is the standard form of unit by PLOS publication.**

**Response:**

Thank you for your suggestion! We have revised the unit. Please see lines 243-244 and 255-256 at the Revised Manuscript with Track Changes.

**L218-223: More semicolons. Why use the semicolon when the sentence can be finished with a period? Also, I don’t follow the use of p-value here and the next paragraph with STN and SOP. Does the p-value represent t-test or LSD comparison of two treatments? If so, this should be clearly stated either here or in the statistical analysis method section. There should be only one p-value for the ANOVA with six treatments.**

**Response:**

Thank you for your suggestion! We have deleted the semicolon. Please see lines 244-250 at the Revised Manuscript with Track Changes. The p-value represent LSD comparison, we have added the statements. Please see lines 230-232, 246, 248-250, and 258-260.

**L223-226: The same problem about semicolon. In addition, these p-values are actually presented in the table, so it is not necessary to repeatedly present in the text. The same problem also happened in the following sections of results, please check and revise.**

**Response:**

Thank you for your suggestion! We have deleted the semicolon and the redundant p-values. Please see lines 250-254 at the Revised Manuscript with Track Changes.

**L230: Change to “STP did not change significantly across treatments”, and delete “p>0.05”. Same for the last sentence and elsewhere in the manuscript.**

**Response:**

Thank you for your suggestion! We have revised the sentence. Please see lines 258-259 and 262 at the Revised Manuscript with Track Changes.

**Also, I wonder why the data for WUE under different treatments were not provided. Did WUE change significantly with N addition and watering?**

**Response:**

The data have been published in another paper (Chen et al., 2021, Biogeosciences, 18, 2859–2870). In addition, the main objective of the present study is to determine the effect of precipitation and atmospheric N deposition on the correlation between N, P and K and between WUE and N, P, K in H. ammodendron. Therefore, the data for WUE under different treatments has not been provided in this manuscript. We have deleted the description of WUE and gas exchange measurement in the Materials and methods Section, and added a statement about the data source of WUE and gas exchange in the manuscript. Please see lines 236-238 at the Revised Manuscript with Track Changes. We have reported in the paper published in BG that WUE changed significantly with N addition and watering.

**L247-251: Still the semicolon problem. The text clearly presented two sets of the results, i.e. the WUE-NPK correlations and the NPK correlations, so why not separate them with a period? Also details of the linear regressions were provided in the figure and again in the Table 2, and once again in the text here. Please only present what you think the most important information in the parentheses and delete the repeated ones. The same problem with the next sentence and sentences in the next two paragraphs.**

**Response:**

Thank you for your suggestion! We have revised the sentence and deleted the redundant information in the parentheses. Please see lines 275-283 at the Revised Manuscript with Track Changes.

**L323: change to “there was no significant correlation”**

**Response:**

Thank you for your suggestion! We have revised the sentence. Please see line 337 at the Revised Manuscript with Track Changes.

**L325-327: This sentence means the same with L322-324. Please delete it.**

**Response:**

Thank you for your suggestion! We have deleted the sentence. Please see line 340-341 at the Revised Manuscript with Track Changes.

**L341-343: This sentence described a result (Table S2), which should be mentioned in the Results. Overall the results of WUE (or gas exchange parameters) were lost from the Results. Does the authors have a good reason for not presenting these results?**

**Response:**

Thank you for your suggestion! However, the aim of the present study is to determine the effect of precipitation and atmospheric N deposition on the correlation between N, P and K and between WUE and N, P, K in *H. ammodondron*. In addition, the results of gas exchanges and WUE have been reported in our paper published in Biogeosciences (Chen et al., 2021, Biogeosciences, 18, 2859–2870). We presented the results of the correlation between N, P, K and gas exchange in S2 Tables to only explain the observed irrelevance between N, P, K and WUE. So we did not present them in the Results section.

**L348: change “and” to “but”**

**Response:**

Thank you for your suggestion! We have revised the word. Please see line 364 at the Revised Manuscript with Track Changes.

**L352: change to “needs confirmation” or “needs to be confirmed”**

**Response:**

Thank you for your suggestion! We have revised the sentence. Please see lines 367-368 at the Revised Manuscript with Track Changes.

**L353: There is no need to start a new paragraph because here presented another reason for the non-significant correlation between WUE and plant NPK.**

**Response:**

Thank you for your suggestion! We have merged the two paragraphs. Please see line 368 at the Revised Manuscript with Track Changes.

**L364-370: These three sentences can be simplified into one sentence. For example: “The correlations between plant NPK varied with water addition levels (Fig. 2), N addition levels (Fig. 3), as well as the interaction between N addition and watering (Fig. 4). There results confirm …”**

**Response:**

Thank you for your suggestion! We have revised the sentence. Please see lines 380-390 at the Revised Manuscript with Track Changes.

**L375: change to “in that case”**

**Response:**

Thank you for your suggestion! We have revised the sentence. Please see line 392 at the Revised Manuscript with Track Changes.

**L380-381: change “on the other hand” to “****in contrast”. Also is there any reference or explanation on this description that plant demands for P is greater than that for N when precipitation increases (the authors did not point out which direction of the change in precipitation)?**

**Response:**

Thank you for your suggestion! We have revised the sentence. Please see line 397 at the Revised Manuscript with Track Changes. We made some mistakes in this description. What we want to express is that *H. ammodendron* in the present study may demand more P than N under precipitation increase. The reason is that the N/P ratio in *H. ammodendron* was mostly higher than 16, which have been mentioned later (lines 366-371). We have revised this description. Please see lines 403-404 at the Revised Manuscript with Track Changes.

**L382-384: Bad grammar. Change to “… indicator of the relative limitation of N** **versus P** **in ecosystems, i.e., N/P ratio less than 14 generally indicates N limitation while N/P ratio greater than 16 suggests P limitation.”**

**Response:**

Thank you for your suggestion! We have revised the sentence. Please see lines 399-402 at the Revised Manuscript with Track Changes.

**L384-385: “almost higher than 16”? Do you mean “mostly higher than 16”? From Table S3, all mean N/P ratios were higher than 16. Also this should be mentioned in the Results section.**

**Response:**

Thank you for your suggestion! We have change “almost” to “mostly”. Please see line 403 at the Revised Manuscript with Track Changes. Also, the result of N/P ratio mentioned here is to explain that the demand for P is higher than the demand for N in *H. ammodendron* in the present study, not the key results of the present study. So we did not present it in the Results section.

**L394: Where did the “interaction of temperature and precipitation” come from?**

**Response:**

Sorry for our mistake. This sentence should be “interaction of N deposition and precipitation”. We have revised it. Please see line 414 at the Revised Manuscript with Track Changes.

**Reviewer #2: This is the second time I have reviewed this manuscript, and I feel the authors have really improved the manuscript and limited the scope of their results to accurately reflect what the data are indicating. but, the standard of English is poor - even in this revision. Providing this is done, the manuscript is in my opinion acceptable for publication.**

**Response:**

Thank you for your suggestion. We have asked an English editing company (Editage) to edit English.
