# Supplementary material for: Changes in precipitation and atmospheric N deposition affect the correlation between N, P and K but not the coupling of water-element in Haloxylon ammodendron
Source: PLoS One. 2021 Oct 22;16(10):e0258927. doi: 10.1371/journal.pone.0258927 (PMC8535465; doi:10.1371/journal.pone.0258927)
Supplement: Attachment — Submitted filename: Responses to the Reviewers.docx [file pone.0258927.s009.docx]

**Journal Requirements:**

**Please review your reference list to ensure that it is complete and correct. If you have cited papers that have been retracted, please include the rationale for doing so in the manuscript text, or remove these references and replace them with relevant current references. Any changes to the reference list should be mentioned in the rebuttal letter that accompanies your revised manuscript. If you need to cite a retracted article, indicate the article’s retracted status in the References list and also include a citation and full reference for the retraction notice.**

**Responses:**

Thank you for your suggestion. We have reviewed our reference list. We have not cited any retracted papers. In the last revision, we have added 1 reference, it is reference [46]. However, we did forget to mention this change in the Responses to the Reviewers of the previous version. Sorry for our mistake in the last revision.

**Additional Editor Comments (if provided):**

**The author had deleted Measurements of gas exchanges and calculations of water use efficiency in Materials and method. However, in L282, there are citations of two equations in Discussion: "According to the definitions of ins-WUE and int-WUE (equations (1) and (2))," Please modify discussion according to Materials and method.**

**Responses:**

Thank you for your suggestion. We have revised the sentence. Please see lines 281-285 at the Revised Manuscript with Track Changes.

**The full comma "." was missing in Line 332.**

**Responses:**

Thank you for your suggestion. We have added the full comma “.”. Please see line 334 at the Revised Manuscript with Track Changes.

**There are some format mistakes in References. In 21, 22, 25, 37, 39, 50, 54 and 55, all Latin name in title should be italic. In 22, genus name should be Artemisia and Caragana. In 50, only the first letter of the first word need to be capital. Please check them carefully.**

**Responses:**

Thank you for your suggestion. We have checked the References and revised the mistake. Please see the References section at the Revised Manuscript with Track Changes.
